# The saga of James Lucett and the process for curing insanity, Part 1 (1811–14): The rise and fall of Delahoyde and Lucett

**DOI:** 10.1177/0957154X231199352

**Published:** 2023-11-08

**Authors:** Leonard Smith

**Affiliations:** University of Birmingham, UK

**Keywords:** Celebrity, cure, patronage, process, quackery

## Abstract

James Lucett, a London clerk, claimed possession of a secret remedy for curing chronic insanity. In 1813, he and the Irish surgeon Charles Delahoyde secured royal and aristocratic patronage to implement their ‘process’ and opened a private asylum. They aroused great public interest after apparently remarkable results with hitherto intractable patients from Bethlem and Hoxton. Delahoyde and Lucett attained brief celebrity, but within a year it was evident that the dramatic recoveries were only temporary. Their venture collapsed in disarray and bankruptcy, and the episode was soon largely forgotten. Delahoyde fled to Ireland, but Lucett managed to re-establish himself in practice. This article narrates the origins, operation and failure of the enterprise. A second article will consider Lucett’s subsequent career.

## Introduction

In 1811, when public anxiety regarding King George III’s mental disorder was at its height, a Bank of England clerk named James Lucett claimed to possess a secret remedy for curing people hitherto regarded as incurably insane. Together with the surgeon Charles Delahoyde, they convinced people in high places that their ‘process’ merited a proper trial. In 1813, with sponsorship from senior members of the royal family and aristocracy, Delahoyde and Lucett opened a house where their methods were put into practice. Great public interest was aroused after their apparently remarkable success with several previously intractable patients drawn from Bethlem Hospital and elsewhere. For a brief period, Delahoyde and Lucett attained great celebrity. However, within about a year, it had become evident that the dramatic recoveries were not being sustained. Their venture collapsed in disarray and mismanagement, and the brief episode was soon largely forgotten (Hunter and Macalpine, 1963: 326; Parry-Jones, 1972: 192–3; Smith, 2020: 94, 215). Charles Delahoyde fled to Ireland, where he became embroiled in greater scandal. James Lucett was able to re-establish himself and continued in private practice for another 20 years. This article narrates the origins, implementation and failure of the project. A linked second part will consider James Lucett’s subsequent interesting and eventful career.

The first two decades of the nineteenth century were particularly noteworthy for the volatile political, social and economic condition of Britain and its people. A combination of wars, trade disruption, industrial upheaval and repressive government policies fostered widespread discontent and public protest (Rule, 2014; Thompson, 1968). The unsettled state of wider society provided the background context for a significant period in the history of psychiatry. Public consciousness regarding the problems associated with insanity reached its height, linked to several notable events. At the forefront was the periodic madness of the King which, when most acute, dominated the news and aroused enormous anxiety throughout the country. This was most pronounced during his first major episode in late 1788 and early 1789. The royal recovery, under the controversial ministrations of Reverend Dr Francis Willis, brought an outpouring of national rejoicing. The King experienced significant relapses in 1801 and 1804, from both of which he gradually recovered within a few months. However, the episode which began in late 1810 proved more serious and enduring. Despite the efforts of the royal physicians and insanity specialists like Drs Robert Darling Willis and Samuel Foart Simmons, the monarch’s descent into chronic incurable insanity was clear. The palpable mood of public gloom was amplified with the installation of the highly unpopular Prince of Wales as Prince Regent (Macalpine and Hunter, 1969: 14–95, 111–37, 143–68).

In addition to his personal sufferings, the King was also subjected to the consequences of other people’s dangerous mental disturbances. In 1786, the hapless attempt by Margaret Nicholson to stab him led to her long-term incarceration in Bethlem Hospital. Bethlem was also the eventual destination for James Hadfield, following his more serious assassination attempt at Drury Lane Theatre in 1800. These cases made clear the severe risks posed by the unpredictable actions of people who felt themselves compelled to act upon unchecked paranoid or grandiose delusions. The most disastrous case was that of John Bellingham, whose perceived grievances led to his fatal shooting of the Prime Minister Spencer Perceval in the lobby of the House of Commons in 1812. Indications of Bellingham’s mental derangement were dismissed by the judge, and he was tried, convicted and executed within a week of the attack (Andrews and Scull, 2001: 215–54; Gillen, 1972; Macalpine and Hunter, 1969: 310–19).

A heightened public awareness of insanity and its depredations gave added impetus to a growing discourse around how the problems might be tackled. At a national level, the efforts of lunacy reformers brought about the crucial parliamentary select committee of 1807 and the passage of ‘Wynn’s Act’ in 1808, thereby initiating what became a national system of county lunatic asylums. The more immediate shortcomings of some existing public and private institutions were thrust into the public domain by the parliamentary investigations conducted in 1815, providing further ammunition for an already aroused court of public opinion (British Parliamentary Papers [BPP], 1814–15; BPP, 1816; Jones, 1972: 54–87; Scull, 1993: 87–90, 115–22; Smith, 1999: 20–36).^1^

The overall public policy response to the management of insanity was clearly important. However, at an individualised level, the problem of finding an effective treatment was more immediate. The conventional modes of medical and surgical treatment were acknowledged to have produced variable results at best, while the newer, more interactive techniques of ‘moral treatment’ were still relatively unproven. There was perhaps a tendency to gravitate toward any new method, however dubious or drastic, that offered some prospect of effecting an expeditious cure, as evidenced by the attention given to Joseph Mason Cox’s circular swing chair (Cox, 1804: 102–36; Dickson, 2018; Hunter and Macalpine, 1963: 595–8; Wade, Norrssell and Presly, 2005). A preparedness to adopt any seemingly promising remedy pervaded all levels and ranks of society, even if its nature was shrouded in mystery. Great possibilities of fame and fortune could be available to any practitioner, whether regular or irregular, who produced a successful cure for the worst cases of insanity.

## The ‘process’

The unlikely story of James Lucett’s acquisition of an alleged secret formula for a means to cure insanity bore all the hallmarks of a classic eighteenth-century quack’s self-promotion tale (Porter, 1989). Lucett was born in about 1768. He received no formal medical training and, when he first came to public attention in 1811, he had been for ‘many years’ a clerk in the Bank of England. He resided in Somers Town, a middle-class suburb of north London (*Morning Post* [*MP*], 24 Aug. 1811; *The Examiner*, 26 Jan. 1812; Anon., 1813a). Lucett’s father had been a former officer in the British military’s ‘Prussian service’ and he spoke several languages. Circumstances led him to provide hospitality to a shipwrecked foreign physician who, while staying in the household, heard of a deranged man in the locality whom the ‘principal medical gentlemen’ of the town had attended without success. The patient was eventually placed under his care and made a remarkable recovery. The anonymous physician subsequently embarked for America, but died on the voyage. Before departing, in gratitude for Lucett’s hospitality, he communicated the ‘valuable treasure’ of his secret treatment method both orally and in writing, advising him to have a son medically educated in order to make full use of it. James Lucett’s older brother was consequently designated for the medical profession, but both he and his father died prematurely, leaving James as ‘sole depositary of the remedy’ (Lucett, 1815: 7–9).

The manuscript remained locked in Lucett’s desk for some considerable time. In a desire to ‘do something’ for his family and to promote the ‘public good’, he eventually turned his attention to it. His ambitions were considerable from the outset. In March 1811, when concerns about George III’s condition were at their height, Lucett contacted the royal physicians to offer them ‘a process that had been successfully practised by a foreign Physician on an Hanoverian Officer, who was labouring under a similar malady to that of his MAJESTY’. The man, who displayed ‘symptoms of an effusion of the brain’, had previously been declared incurable by ‘the Faculty’. The King’s physicians doubtless regarded the proposal as merely another piece of quackery to be ignored. Undaunted, in August 1811 Lucett advertised in a London newspaper, seeking intercession by the Queen. He wanted the royal physicians to ‘make an enquiry into the merits of the case’, contending that adoption of the ‘process’ would be ‘the means of restoring our beloved Sovereign once more to the society of his august family and loving subjects’ (*MP*, 24 Aug. 1811; Lucett, 1815: 9–10).

Lucett quickly realised that assertions alone would not suffice and that some practical demonstration was required. He also recognised that his own credibility was impaired by the absence of a formal medical qualification, prejudicing the possibilities of finding suitable patients. He decided to join forces with a professional medical man, for the purpose of implementing a trial of the ‘process’. The first person to come forward was Elias Tardy, a surgeon living near Russell Square. Their chosen patient was Mr Morgan, editor of the *Dublin Correspondent*, a man of 44 years, ‘inclining to corpulency’ as well as intemperance. He had been insane for 18 months, and was ‘attended by some eminent gentlemen in that practice’. The case ‘had become one approaching to idiotism blended with melancholy’. He experienced very restless nights, ‘getting up frequently to sing’, and often needed to ‘go to stool’ (Lucett, 1815: 10; Tardy, 1813: 125).

The treatment began at Lucett’s house in the evening of 23 September 1811. It evidently involved extended immersion in a warm bath, followed by simultaneous application of a ‘considerable column’ of warm water to the shaven head, producing slight concussion. Morgan slept profoundly for over 12 hours, apart from being awakened for meals, after which he was in a much calmer and more lucid state. The improvement was maintained for three days and nights. Convinced that the ‘process’ had already succeeded, Lucett announced it in the *Morning Post* on 27 September, under the tantalising heading ‘State of the King’. Stressing the eminence of the ‘professional man’ treated, Lucett’s confidence knew no bounds as he proclaimed: ‘I will stake my existence that I relieve the King in twenty-four hours, and finally recover his Majesty within a fortnight’ (*Lancaster Gazette*, 5 Oct. 1811; *MP*, 27 Sep. 1811; *The Examiner*, 2 Oct. 1814; Tardy, 1813: 125–7).

However, Lucett had been rather premature. On the day of publication, Morgan became ‘somewhat more troublesome’ and resumed singing. The ‘operation’ was repeated the next day, followed by several relatively settled days. By 1 October, the ‘irrational words’ had ceased, his dress was correct, his behaviour appropriate and he wanted to go out walking with his wife. According to Elias Tardy, some residual symptoms remained at this stage. These were glossed over by Lucett, who submitted a highly positive report to the Prime Minister, Spencer Perceval, which Tardy endorsed. Lucett anticipated that Perceval would bring pressure to bear on the King’s physicians to take up his ‘wonderful remedy’. He later claimed to have met personally with Perceval, and on his advice wrote to Dr Robert Darling Willis offering to submit his means of cure to the royal physicians, but they continued to ignore his approaches (*Lancaster Gazette*, 5 Oct. 1811; *MP*, 27 Sep. 1811; *The Examiner*, 26 Jan. 1812; Tardy, 1813, 125–6).^2^

A more serious concern then presented itself in the treatment of Morgan. An ‘incautious and sudden increase of the temperature of the bath’ led to the flare-up of a pre-existing haemorrhoidal condition, precipitating a ‘considerable and dangerous discharge of blood’. Lucett and Tardy hoped initially that this might prove in some way therapeutic. However, after three days, Morgan’s mental condition deteriorated and he displayed clear indications of ‘fatuity’ and loss of memory. Tardy now insisted that the treatment be discontinued, although he later acknowledged that its ‘moderate application’ had brought ‘unequivocal symptoms of amendment’. Presumably worried about the effects on his professional reputation, he parted company from Lucett shortly afterwards, although there was no apparent public disagreement. Indeed, Tardy subsequently opened his own private ‘establishment’ at Enfield, where he intended to try a modified version of the ‘process’ on Morgan and other patients (Royal College of Physicians Archives [RCPA], 1813; Tardy, 1813: 127).

Despite this setback, Lucett remained outwardly confident, clearly mindful of the commercial possibilities of the ‘process’. In November, while acknowledging disappointment in his endeavours to benefit the King, he offered his services to members of the public ‘afflicted with the . . . DREADFUL MALADY’ of insanity. In particular, he targeted those ‘who may be deemed incurable by the Faculty’, a proviso that became a standard feature of his propositions. Shortly afterwards, Spencer Perceval belatedly responded to Lucett’s report regarding the trial of the ‘process’ on Morgan, advising that ‘he cannot venture to recommend to his MAJESTY’s Physicians, to permit any experiment to be made with regard to his MAJESTY’s health’, which they would not approve. In a characteristically forthright manner, Lucett demanded that the physicians should neither approve nor disapprove until they had ‘examined the nature of the remedy, of which they are now in complete ignorance’, insisting that it was ‘not the recipe of a Quack, but of a regular Medical Practitioner’. They were ‘not doing their duty’ by refusing to consider any course other than their own, which they admitted had not succeeded (*MP*, 11 Nov. 1811; *The Examiner*, 26 Jan. 1812).

Lucett’s contentions now began to attract vocal critics, with barely concealed inferences that he was merely a quack. Referring to the ‘*infallible* remedy he says he possesses for the cure of madness’, one anonymous commentator accused Lucett of a ‘manifest falsehood’ in asserting that Morgan had been cured by ‘this recipe’. His disorder had recently become ‘so violent, that he was obliged to be constrained by the strait waistcoat’. Although repeatedly subjected to Lucett’s ‘infallible’ remedy, he had never ceased to be insane and was on 28 January ‘in the most afflicting state of the malady’. Any certificate of Morgan’s cure sent to Spencer Perceval must have been ‘a most infamous fabrication’. The same writer repeated the criticisms, with even greater vehemence, in 1814 (*The Examiner*, 2 Feb. 1812; 2 Oct. 1814).

James Lucett recognised that he needed another suitably qualified associate in order to disseminate his curative process to a sceptical public, but it proved difficult to find a medical man prepared to be instructed by someone outside the profession. Nevertheless, during 1812, after ‘many applications and rebuffs’, he encountered a 39-year-old ‘regularly educated’ Irish surgeon, Charles Delahoyde, who had previously worked at the Foundling Hospital. Lucett found him ‘candid, generous and intelligent’, and he ‘seemed to be the right man’. However, Delahoyde had a rather chequered history, having been in recent financial difficulties which led to a spell in the insolvent debtors’ prison during 1811. Whether Lucett was ignorant of Delahoyde’s embarrassed circumstances, or chose to overlook them, is questionable (*London Gazette*, 14–17 Sep. 1811: 1826; Anon., 1813a: 385; Lucett, 1815: 10–11).

Delahoyde had no particular experience of working with mentally disordered people, but was undoubtedly attracted by the prospects of great financial reward if the secret treatment process proved successful. Both men recognised the need to demonstrate its efficacy before they could attract paying patients. Their first trials, carried out on pauper lunatics and others not confined in an asylum or madhouse, made no impression upon the public. Initial attempts to obtain a patient from a public lunatic hospital for their experiment were rebuffed. However, in the summer of 1812, the fortunes of Delahoyde and Lucett began to change. They attracted the notice of the Duke of Kent, brother of King George III. The Duke was doubtless anxious to seize upon any possible means to restore the sovereign’s health, however remote. He exerted royal influence on the reluctant governors of Bethlem Hospital to submit a patient for trial of the ‘process’ (Lucett, 1815: 12–13; see also Bethlem Royal Hospital Archives [BRHA], 1812: House Committee Minutes [ HCM-20], 19 Sep.).

The person selected, William Harrison, had a military background and was formerly the Duke of Kent’s bandmaster. Having been declared incurable by the Bethlem physician Thomas Monro, he had been confined for four years and was regarded as one of the hospital’s ‘most outrageous’ patients. On 30 September, Harrison was taken to a house occupied by Lucett at West Ham. After the ‘process’ was implemented on the first evening, he slept soundly and spent a settled first day without the need for restraint. On the third day Harrison was ‘perfectly rational’, eating well and playing his clarinet. He was reconciled with family members, whom he had previously regarded with disdain. On the fifth day he attended church with Delahoyde’s family, conducting himself ‘with propriety’, spending the rest of the day with his wife and playing music in the evening. On 11 October, he and Mrs Harrison visited their children in Chelsea. Four days later he was taken to Kensington Palace, where he conversed with the Duke of Kent and expressed his gratitude, before visiting some old comrades at the barracks. On 16 October he performed on his clarinet at the Duke’s country house. After four weeks, Harrison was considered fully recovered and discharged home. Delahoyde and Lucett were subsequently granted 200 guineas by the Lords of the Treasury for their cure of Harrison (BRHA, 1812, HCM-20, 26 Sep., 3 Oct.; Sheffield City Archives [SCA], 1813, WWM/F/64/190; *MP*, 31 Aug. 1816; Anon., 1813a: 385–6; Lucett, 1815: 13–15).

Flushed with this evident success, and fortified by royal support, Delahoyde and Lucett rented a substantial house at Sion Vale, near Brentford, west of London. They received their second Bethlem patient, John Moon, a private in the Marines, on 21 December 1812. He had been chained and handcuffed for nearly a year and was considered one of Bethlem’s most violent patients. Once again the Duke of Kent was instrumental. The Lords Commissioners of the Admiralty ordered that ‘he be delivered up to the care of Mr De la Hoyde for the purpose of attempting his Cure’. Moon arrived at Sion Vale in an ‘outrageous state’. The ‘process’ was carried out on the first evening, producing a dramatic reduction in his pulse rate. He slept soundly and was tranquil the following day. By the seventh day Moon was ‘convalescent’ and on 29 December he took a walk with Lucett, stopping to converse with the captain at Hounslow barracks. Two days later Lucett took him into London. At a public house opposite Bethlem, Moon met some of his erstwhile keepers, who were astounded at the change that had occurred. They visited the Naval Transport Board offices, where Moon was examined by its physician Dr John Harness, who was also ‘astonished’. Moon remained at Sion Vale for several weeks, working in the garden. On 22 April 1813, the Admiralty acknowledged that he had ‘recovered his senses’ and could be sent to the *Batavia* hospital ship at Woolwich for a month’s probation, prior to returning to naval service (BRHA, 1812, HCM-20, 12, 19 Dec.; National Maritime Museum [NMM]: ADM/ET/58, 5 Dec. 1812; ADM/ET/59, 22 Apr. 1813; SCA, 1813, WWM/F/64/190, 8 June; Anon, 1813a: 386–7).^3^

While Moon was recovering at Sion Vale, a high-profile patient arrived on 11 April. Mrs Elizabeth Lancaster, wife of Joseph Lancaster the prominent educational reformer, had been insane for eight or ten years and certified as incurable by ‘three eminent physicians’. She too was placed with Delahoyde and Lucett following recommendation by the Duke of Kent. After undergoing the ‘process’, her condition improved markedly. On 25 April, Delahoyde and Lucett advised the Duke that Mrs Lancaster had been successfully reintroduced to her estranged daughter and other family members. After six weeks her husband and their young child were permitted to stay with her at Sion Vale. Her recovery continued and she attended meetings of the Lancastrian Society. At an annual meeting of Quakers she behaved with ‘perfect correctness’. After several extended visits to her home in Tooting, Mrs Lancaster was discharged in June 1813. In their ‘gratification’, both she and her husband gave approval for the case to be publicised (SCA, 1813, WWM/F/64/190, 8 June; Anon, 1813a: 387; Lucett, 1815: 18).

## In the ascendant

By the spring of 1813, there was growing public awareness of the apparently remarkable results achieved by Delahoyde and Lucett. Hopes of an effective cure for insanity were encouraged by the widespread coverage in newspapers as well as literary and medical journals. Reports of people whose cases had been declared hopeless by respected physicians, but were restored by means of a secret ‘curative process’, proved intoxicating. According to one commentator, the public regarded the cures achieved as ‘the wonder of our age’. The infectious enthusiasm permeated through to the highest echelons, embracing royalty and leading members of the aristocracy, as well as politicians, scientists and philanthropists (*Bath Chronicle*, 18 Mar. 1813; *Oxford University and City Herald*, 12 June 1813; *Staffordshire Advertiser*, 12 June 1813; *Bury and Norwich Post*, 16 June 1813; *Suffolk Chronicle*, 19 June 1813; *Norfolk Chronicle*, 19 June 1813; *The Examiner*, 26 Dec. 1813). Of course, scepticism was expressed in certain quarters. The madhouse proprietor Thomas Bakewell derided the report of cures by Delahoyde and Lucett as a ‘premature puff’, for nobody had yet been discharged fully cured, and mere ‘lucid intervals’ did not suffice (*Staffordshire Advertiser*, 19 June 1813).

The Duke of Kent had clearly been impressed by the progress of the three patients whom he had recommended. He enlisted the participation of his brother the Duke of Sussex, and a subscription was launched to enable evaluation and propagation of the new treatment method. As well as the Royal Dukes, subscribers included the Duke of Bedford, Earl Fitzwilliam, Lord Dundas and the President of the Royal Society, Sir Joseph Banks. They all formed part of a committee of 15 men, presided over by the Duke of Kent, along with the Earl of Cork, Lord Milton, Viscounts Melville and Palmerston, and the naval physician Dr John Harness. It was envisaged that a successful outcome could lead to the establishment of a permanent society to implement the treatment (SCA, 1813).

By the end of May 1813, the Committee had developed a clear structure for the project’s operation and for conducting the enquiry. A ‘convenient receiving-house’ would be provided for 10 patients, under the care and management of Delahoyde and Lucett. The patients were to be selected by the committee, following approval by Charles Delahoyde in his capacity as surgeon, and they could remain free of charge for three months, or longer if ‘necessary for their restoration’. Committee members might visit at their convenience, although Delahoyde could refuse this at ‘improper’ times. Records would be kept regarding the progress of ‘cure’, and regular reports published. Expenses and remuneration for Delahoyde and Lucett would be covered by monies subscribed. Sion Vale’s legal status as the ‘receiving-house’ was regularised by acquisition of a license from the Royal College of Physicians, under the Regulation of Madhouses Act, 1774 (RCPA, 1813; SCA, 1813, WWM/F/64/190, 31 May; *Bell’s Weekly Messenger*, 20, 27 June 1813).

The Committee wanted to ensure that investigations of the ‘process’ provided ‘the severest possible test’. The ‘superior judgment and ability’ of Dr John Harness was enlisted for selection of patients for ‘the experiment’. On his advice, they would be chosen from ‘government asylums’, where ‘persons in the public services’ had been placed under the authority of medical certificates provided by Harness or ‘other medical men of eminence’. The ‘Government receiving-house’ at Hoxton was specifically selected. This was in fact Hoxton House, the large private madhouse operated by Sir Jonathan Miles, a significant proportion of whose patients were placed under contract from the Navy. Seven members of the Committee, including the Royal Dukes, Earl Fitzwilliam and Harness, met there on 7 June, to select patients. Harness produced a list of 146 seamen and marines in the house, 94 of whom had been declared incurable by the naval inspector Dr John Weir, the Bethlem apothecary John Haslam, or the Hoxton House surgeon James Sharpe. The Committee saw each person considered incurable and, with assistance from Delahoyde and information from Miles and his keepers, chose ‘such of them as were deemed proper for the experiment of the plan’. Nineteen men were short-listed, from whom Delahoyde selected ‘such as in his judgment were within reach of his process and most fit to answer the object in view’ (Anon., 1813b: 4–7).

Arrangements were made with Delahoyde and Lucett regarding terms for initially receiving four patients and others subsequently. It was apparent that Charles Delahoyde, as the qualified surgeon, was being accorded precedence in the proceedings, even to the extent of it being described as ‘his process’, and James Lucett was becoming uneasy about the possibility of being side-lined. Other concerns also began to emerge. On 1 July, Delahoyde’s evident intent to maximise his personal profits led Dr Harness to express concerns to Earl Fitzwilliam that he had taken advantage of advertisements placed by the committee and ‘had begun to fill his House with private Patients’, before establishing ‘the Validity of his Claim to public attention’. The presence of these private patients contributed significantly to a lack of capacity at Sion Vale, resulting in delays to the removal of people from Hoxton. Only by the end of July had the agreed number of four been received, almost two months after the patients were interviewed (SCA, 1813: WWM/F/64/196, 197, Harness to Fitzwilliam [1 July]; WWM/F/64/200, Delahoyde to Fitzwilliam [3 Aug.]; Lucett, 1815: 16–18).

However, any unease regarding these concerns was set aside as the trials gathered pace, with early indications of success. The first two patients, John Braily and Richard Harris, were received on 27 June. Harris was soon returned to Hoxton House, Delahoyde having concluded that he was unsuitable for the ‘process’ due to pre-existing brain damage, a fact disputed by Hoxton’s surgeon, James Sharpe. Daniel O’Keefe was admitted on 16 July, followed by William Matters and James Cardiff on 30 July. Braily, Harris, Matters and Cardiff were all naval patients who had spent a year at Bethlem and then several years at Hoxton. The exception was O’Keefe, a former army quartermaster and a Chelsea Pensioner, who had been cared for by his wife at home in Berkshire after periods at Bethlem and Hoxton, and was admitted following examination by the Royal Dukes and Harness (SCA, 1813, WWM/F/64/197, Harness to Fitzwilliam [1 July]; National Archvies [TNA], 1813, ADM 102/232 [30 July]; *The Examiner*, 26 Dec. 1813; Anon., 1813b: 7–13).

John Braily, when interviewed by the Committee, was ‘heavily chained and handcuffed’ and ‘without breeches’. Appearing ‘more violent, if possible, than the rest’, he was singled out by the Duke of Sussex as a ‘proper, and desirable subject to undergo the Experiment’. He was conveyed to Sion Vale, covered only by a ‘horse-cloth’. Although calm on arrival, after three hours ‘he became so violent as to break asunder the apparatus prepared by Mr Delahoyde for his confinement’ during the ‘operation’, requiring postponement of the ‘curative Process’ for two days. Dr Harness visited Braily on 29 June, measuring his pulse before and afterwards. From a peak of 108 per minute, it was reduced after three-quarters of an hour to 76. More significantly, Braily was ‘in every Respect more calm’. He told Harness that ‘he had undergone a severe Operation: that he had given the Gentleman [alluding to Mr D] much trouble, but, hoped that he should soon be better’. On 1 July, Delahoyde recorded that Braily was amusing himself by walking in the garden and playing draughts. On 4 July, Harness spoke to him in the garden. Braily reported feeling rather weak, but expressed gratitude to Delahoyde for the great benefit he had received, stating that ‘he would ever consider him as his greatest friend’ (SCA, 1813: WWM/F/64/198, Delahoyde to Fitzwilliam [9 July]; WWM/F/64/199, Harness to Fitzwilliam [19 July]; Anon., 1813b: 7–10).

Subsequent visits to Sion Vale by Harness, the Royal Dukes and other committee members brought reports of Braily being ‘perfectly composed’ and continuing to progress. Harness told Earl Fitzwilliam that he was ‘at a Loss to account for the sudden Effects produced by the Means employ’d’. He was unsure of ‘the Probability of the Permanency to the Patient’, which could ‘only be ascertained by Passage of Time, assisted by great Temperance and Regularity of Life’. On 7 August, Delahoyde reported that Braily was in a convalescent state. Meanwhile, newspaper reports around the country spoke excitedly of Delahoyde having ‘performed one of his miraculous cures’ on one of the ‘wildest’ and ‘most ungovernable’ patients in Hoxton, while others had also been cured by a means that remained a ‘perfect secret’ (SCA, 1813, WWM/F/64/199, Harness to Fitzwilliam [19 July]; Anon, 1813b: 10; *Caledonian Mercury*, 29 July 1813; *Hereford Journal*, 4 Aug. 1813; *Ipswich Journal*, 31 July 1813; *Liverpool Mercury*, 6 Aug. 1813).

The continuing secrecy fuelled growing speculation regarding the ‘process’ itself. It was suggested in some medical quarters that Delahoyde was having ‘recourse to exhaustion’, which he denied. A contributor to a Bristol newspaper claimed that the treatment consisted mainly of ‘the application of warm water as a general bath’, followed by ‘warm affusions’ on the shaven head ‘by means of a considerable column of water’, thereby producing ‘a slight concussion’. This was sometimes augmented by a ‘cold affusion’, and the remedies combined to produce quiet, a ‘propensity to sleep’ and, if continued long enough, a loss of consciousness. That description approximated closely to the method delineated by the surgeon Elias Tardy (who had earlier worked with Lucett) in a letter to the *Medical and Surgical Journal* dated 20 July 1813. He placed it in the context of long-established water treatments, observing that a ‘bath of very high temperature almost always allays the paroxysm of mania’, concluding that the benefit of applying a warm or cold column of water ‘arises from the concussion produced’ (*Bristol Mirror*, 14 Aug. 1813; *Caledonian Mercury*, 29 July 1813; Tardy, 1813: 127–8).

The other two naval patients from Hoxton, William Matters and James Cardiff, were not as disturbed as John Braily following their reception at Sion Vale, but they displayed similar initial dramatic improvements. They both arrived on 30 July, and undertook the ‘curative process’ on their first evening, sustained significant reductions in pulse rate, then fell into a sound sleep before waking up in a tranquil state. Matters was ‘sane’ and working in the garden three days later, while Cardiff spent his second day assisting the cook in preparing dinner for the house. On 3 August, Delahoyde assured Earl Fitzwilliam that they would be restored within two months, and on 7 August he recorded that both were already in a convalescent state. Daniel O’Keefe, who had arrived two weeks previously, showed slower improvement. He experienced a comparable reduction in his pulse rate after the ‘process’ and then slept soundly. After several days he too began working in the garden, though he continued to display poor recollection despite Delahoyde’s insistence that his memory was improving (SCA, 1813: WWM/F/64/200, Delahoyde to Fitzwilliam [3 Aug.]; WWM/F/64/201, Harness to Fitzwilliam [8 Aug.]; Anon., 1813b: 10–13).

In mid-August 1813, optimism about the ‘process’ and its prospects remained high. However, Dr Harness had started to have reservations, not without grounds. John Moon, who left Sion Vale in April for his probationary period on the *Batavia*, was subsequently readmitted. By late June, he had again recovered. It was deemed unnecessary ‘to continue Moon any longer in confinement’, and it was agreed that he should be discharged from naval service according to his wishes. In late July, he was escorted back to his native Wiltshire, at a time of ongoing unrest in the south-western woollen manufacturing districts. On arriving at Trowbridge, Moon was ‘triumphantly received by a numerous Banditti, as their former Captain, or Leader’. Fears about the possible risks to public safety were subsequently ascertained to be largely groundless. However, by the end of August Moon had been ‘taken into Custody and placed in the Care of a Person, who takes charge of Lunaticks at Devizes’ (NMM, 1813, ADM/ET/59, 30 June; SCA, 1813: WWM/F/64/190, 8 June; WWM/F/64/201, Harness to Fitzwilliam [8 Aug.]; WWM/F/64/203, Harness to Fitzwilliam [28 Aug.]).

Delahoyde and Lucett were presumably unaware of these developments when, on 3 August, ‘Delahoyde and Co.’ petitioned the Lords of the Admiralty for payment, stating that they had ‘applied their process & performed a cure’ and that Moon had been ‘restored to his Friends’. They claimed nearly £100 for ‘ingredients & the curative means’ and a similar sum for board, lodging and attendance. The Admiralty Office advised the naval Transport Board that they did not think the public should bear the expense and ignored the ‘enormous’ demand (NMM, 1813, ADM/ET/59, 3, 4 Aug.). As the Transport Board’s physician, Dr Harness would have been apprised of these circumstances. An interesting aspect of the petition was that the address given for ‘Delahoyde and Co.’ was 16 Albemarle Street, off Piccadilly. This was the home of Richard Troward, a prominent London attorney who had by this time been installed as Secretary to the Committee, and whom Lucett later accused of being in league with Delahoyde in some questionable dealings (NMM, 1813, ADM/ET/59, 3 Aug.; SCA, 1813, WWM/F/64/204, Troward to Fitzwilliam [10 Sep.]; Lucett, 1815: 16–27).

Harness’s anxieties were increased by a visit to Sion Vale on 10 August, when he encountered John Braily walking in the garden, ‘but not in the quiet composed state I had been accustomed to find him in’. He was ‘particularly talkative’, complaining about his situation and ‘constantly annoyed by numerous People’, saying that ‘he found his head becoming much confused’. At Harness’s behest, Delahoyde applied the ‘curative Process’ again, claiming that Braily derived ‘considerable Relief’. When Harness returned on 25 August, Braily was more settled but still speaking rapidly. Of the other patients, he noted that Daniel O’Keefe had not made ‘any Progress to amendment’, though both Matters and Cardiff were ‘considerably improved’. For the time being, Harness was satisfied (SCA, 1813: WWM/F/64/190, 8 June; WWM/F/64/201, Harness to Fitzwilliam [8 Aug.]; WWM/F/64/201, Delahoyde to Fitzwilliam [8 Aug.]; WWM/F/64/203, Harness to Fitzwilliam [28 Aug.]).

Delahoyde and Lucett remained extremely positive about the results of their interventions. On 13 September they advised that Matters was ‘restored and fit to be discharged’ and Cardiff would soon be ‘perfectly restored’, while Braily and O’Keefe were daily progressing toward ‘restoration’. The committee, including Harness and the Duke of Kent, met at Sion Vale two days later and their report was more circumspect. They acknowledged that Matters showed no symptoms, but observed that Cardiff was ‘not wholly free from the influence of the particular cause to which he attributes his complaint’. Although Braily and O’Keefe appeared calm, the latter’s ‘recollection’ was still ‘much impaired’. Nevertheless, the committee declared on 27 September that so far ‘their hopes have not been disappointed’. In a widely circulated pamphlet, they announced an extension of the experiment by accepting more patients, and they solicited further subscriptions (Anon, 1813b: 16–18).

Delahoyde and Lucett were now flushed with confidence regarding their prospects. In late September they transferred the patients from Sion Vale to ‘extensive premises’ on Great Ealing Green. Nearly all, whether public or private, were stated to be ‘either convalescent or in a progressive state of recovery’. Both men were clearly intent on maximising their lucrative private custom. Towards that end, they sought some official endorsement of the ‘process’. On 28 September, they met with the Dukes of Kent and Sussex, accompanied by Dr Harness, and ‘under a pledge upon honour of secrecy’ communicated ‘their process for restoring insane persons to sanity’. The Royal Dukes and Harness signed a paper confirming that they considered it safe and could not cause injury to the patients (*MP*, 24 Sep. 1813; Anon, 1813b: 19–20). This meeting, and the document produced, would later be widely cited.

Towards the end of October, Delahoyde and Lucett pronounced William Matters and James Cardiff ‘restored to Sanity’. This was communicated to the Admiralty by Richard Troward, the committee’s secretary, whose role was becoming increasingly significant. The Lords of the Admiralty advised Troward that they wanted Dr Harness to examine the men and confirm that they were ‘cured’. He saw them at Ealing House on 3 November, and considered them ‘in a fit state to be discharged’. However, exercising some caution, he recommended that they be sent to the *Batavia* Hospital Ship for a probationary period, the normal practice for naval patients discharged from Hoxton or Bethlem before return to service. Harness was more pessimistic about the prospects for John Braily, placed with Delahoyde and Lucett in late June. The initial benefit received had not been sustained. On several visits, Harness found him ‘in so deranged a state of mind as not to bear to converse on certain Points without its producing considerable incoherence’. He despaired about Braily benefiting further from the ‘Process’ and recommended his return to Hoxton (SCA, 1813: WWM/F/64/205, Barrow to Harness (30 Oct.); WWM/F/64/206, Harness to Croke [9 Nov.]).

Delahoyde and Lucett chose to disregard Harness’s reservations. On 22 November they informed the Lords of the Admiralty that both Cardiff and Matters were ready to be conducted to the *Batavia*. Moreover, they considered John Braily ‘so well, that in our opinion, he might accompany them’. Alternatively, they suggested that he remain a few weeks longer to enable further examination by Harness. Harness was clearly taken aback and, in an appended note, he reiterated that Braily ‘really was perfectly & violently deranged’ when last seen. On 26 November Cardiff and Matters were transferred to the *Batavia.* Although Braily was not sufficiently recovered, he was ‘well enough to work with the outdoor servants and mix with them’. It was agreed that he could remain at Ealing for a few more weeks (SCA, 1813: WWM/F/64/207, Delahoyde and Lucett to Croker [22 Nov.]; WWM/F/64/208, Troward to Fitzwilliam [1 Dec.]; WWM/F/64/209; Harness to Fitzwilliam [14 Dec.]).

Richard Troward, now acting as chief intermediary and cheer-leader, had laid aside any doubts and presented a highly optimistic account to Earl Fitzwilliam on 1 December, 1813. After informing him of the discharge of Cardiff and Matters, and the improvements allegedly being shown by Braily, he cited Delahoyde and Lucett’s ‘very favourable’ report on Daniel O’Keefe, whom Dr Harness had earlier deemed not to be making progress. Troward anticipated O’Keefe’s discharge once Harness saw him again. He reported enthusiastically on several private patents. Harrington, ‘the celebrated Oboe performer’, appeared ‘perfectly recovered’ after nine or ten weeks. Mr Currell from Morpeth, who was a relative of Lord Collingwood and present at the Battle of Trafalgar, had improved rapidly after six or seven weeks and a ‘perfect recovery’ was expected within three months. A lady named Fisher was a ‘still stronger instance of the efficacy of the process’. Having been deranged for ‘6 or 7 years’, her improvement was such that her husband expected she would ‘shortly be restored to him’. Troward’s optimism appeared boundless. He was present when Delahoyde and Lucett communicated their ‘secret’ to the Royal Dukes and Harness, ‘from which time my confidence has greatly increased and now am free from a shadow even of doubt, as to the advantage & efficacy of the process’. He even dared hope that the King himself might benefit, ‘but whether it will ever extend to Windsor is another consideration – if it shd not, it is much to be lamented’ (SCA, 1813, WWM/F/64/208, Troward to Fitzwilliam [1 Dec.]).

The public celebrity of Delahoyde and Lucett was now at its height. They sought to capitalise on all the attention and publicity, proclaiming in mid-December that ‘the success of their process has exceeded the most sanguine expectation’. It had been officially declared ‘free from a possibility of injury to the Patient’, and the ‘restorative merits’ were known to the relatives of recovered patients and to ‘Gentlemen of the Faculty of responsibility’. Following the departures from Ealing House of several people ‘perfectly restored to sanity’, there were now vacancies for more patients. Payment rates were to be ‘governed by circumstances’, and it was even promised that no payment would be required ‘unless cure be effected’ (*London Courier and Evening Gazette*, 14 Dec. 1813; *Morning Chronicle*, 13 Dec. 1813). The profile of Delahoyde and Lucett and their ‘process’ was now so high that they and their ‘invention for the cure of insanity’ had even become the object of political satire, with a quip that the new Bethlem Hospital under construction would not be needed, private madhouses would become unknown, and suicide ‘banished from this happy country’ (Anon., 1813c). In reality, the end of 1813 was the high point of their endeavours, with expectations raised to a level that would be extremely difficult to maintain.

## Failure and recrimination

By the beginning of 1814, clear signs were emerging that Delahoyde and Lucett had over-reached themselves. In their zeal to demonstrate that they had perhaps achieved the supposedly impossible cure for madness, and to reap commensurate financial rewards, they had expanded the operation too quickly and made extravagant claims that would always be vulnerable to disproof. Within a few months, the venture had collapsed, the ‘process’ was discredited and those associated with the experiment were left embarrassed or worse. Charles Delahoyde had fled the country and James Lucett was left to face the critics and the creditors, while various doubtful characters sought to profit further from the remains of the business.

Richard Troward’s role had become increasingly prominent. His reputation had been built partly from participation in a number of high-profile legal cases over a long period, including the impeachment of Warren Hastings. He had also specialised in parliamentary practice, and gained considerable expertise in this field. For a time he exercised much influence as an election agent, and between 1795 and 1800 was proprietor of the notorious ‘rotten borough’ of Ilchester in Somerset, for which he reputedly paid an enormous sum (*Bath Chronicle*, 17 June 1790, 12 Apr. 1792, 26 Dec. 1793; *Hibernian Journal*, 24 July 1780; *Hampshire Chronicle*, 9 Apr. 1781; *Kentish Gazette*, 15 May 1787; Ginter, 1967: 26–7; Thorne, 1986: 347–8; Troward, 1790). However, Troward’s once prosperous financial circumstances had deteriorated considerably and he was in difficulties when he learned of the success achieved by Delahoyde and Lucett. According to Lucett, ‘it became his object to avail himself of the opportunity which he thought he espied of repairing a shattered fortune’. The sequence of events whereby he became directly involved is not entirely clear, nor whether he was appointed secretary to the Committee before or after he ‘wormed himself into the confidence’ of Charles Delahoyde. Similarly, the level of his financial involvement is uncertain, though Lucett later maintained that Troward agreed to advance 3,000 guineas, enabling them to take the house at Sion Vale (Lucett, 1815: 16–19).

Signs that all was not well appeared during the latter part of 1813. On September 28, the day before Delahoyde and Lucett’s disclosure to the Dukes of Kent and Sussex of their ‘Secret’, they informed the Committee that ‘they could not return home that night for the want of £200’, as a writ for bankruptcy had been taken out against them. Those present, who included the Royal Dukes and Harness, reluctantly agreed to advance the money. This was clearly not the end of the story, judging from the letter sent by Troward to Earl Fitzwilliam on 1 December, expressing his unbounded confidence in the efficacy of the ‘process’. In a final paragraph, he advised that Delahoyde and Lucett’s ‘pecuniary difficulties’ were ‘distressing’, due largely to the expenses associated with the move to Ealing House. Consequently, they were compelled to ‘keep out of the way . . . to the very great interruption of their business’. Although it was initially ‘a loosing [*sic*] concern’, Troward was convinced that ‘now the prospect of success is flattering, provided they keep their liberty’. He suggested that ‘a thousand Pounds wd put every thing into comfort’, and offered to assist as far as his ‘humble means’ would allow. Being also also an art dealer and collector, Troward claimed to possess a picture reputed to be ‘the finest Landscape in the Kingdom’, for which he paid 1,600 guineas, and was prepared to sell this for ‘the success of the process’ (SCA, 1813, WWM/F/64/208, Harness to Fitzwilliam [1 Dec.]; SCA, 1815: WWM/F/64/2/216, Smith to Dent [10 Mar.]; Dent to Fitzwilliam [27 Apr.]; *MP*, 17 Apr. 1807; Troward, 1807).

These mounting financial concerns were only one aspect of the difficulties which began to surround Delahoyde and Lucett. In late December, a letter addressed to ‘Their Royal Highnesses the Dukes of Kent and Sussex’ appeared in *The Examiner*, a radical campaigning newspaper. The author was James Birch Sharpe, the surgeon to Hoxton House. He advised the Dukes that, in their quest to promote ‘the amelioration of mankind’, they had been subjected to a ‘most daring imposition’ and were victims of ‘the dangerous designs of Empirics’. Sharpe cited several examples where Delahoyde and Lucetts’ claims of cures were unfounded. An early patient, Mrs Lancaster, was not cured but ‘now labouring under her unfortunate malady’. John Moon had recovered, relapsed and was readmitted before being discharged back to Wiltshire, after which ‘his insanity returned with all its former severity and with unabated fury’, and he was now ‘confined in a lunatic house’. Sharpe alleged that Delahoyde and Lucett were charging ‘enormous sums’, and their claims to cure ‘all kinds of insanity’ were a ‘vain boast’. He hoped the Dukes would realise ‘the consummate quackery of this wonderous plan’ (*The Examiner*, 26 Dec. 1813).

However telling Sharpe’s attack was, the opinion of Dr John Harness regarding the curative attributes of the ‘process’ ultimately carried most weight. The discharge of William Matters and James Cardiff to the *Batavia* at the end of November had marked one of the enterprise’s high points. However, disappointment followed when Harness visited them at Woolwich on 25 January, 1814, accompanied by Dr John Weir, who provided medical oversight to the naval patients at Hoxton. Cardiff, though in an ‘inoffensive state’, expressed elaborate delusional ideas regarding valuable property that he claimed to own in County Wexford. Matters appeared ‘equally deranged, as when at Hoxton’ and ‘extremely incoherent’. In early February, Mr Robertson, surgeon to the *Batavia*, reported that Cardiff was ‘so violently insane that he was obliged to confine him’, while Matters remained very deranged. In consequence of this and the annoyance caused to other patients, both men were ordered back to Hoxton. Drs Harness and Weir had also visited John Braily at Ealing House, on 18 January, and found him too in a ‘highly deranged state’ (SCA, 1814: WWM/F/64/210, Harness to Fitzwilliam [2 Feb.]; WWM/F/64/211, Harness to Fitzwilliam [3 Feb.]; WWM/F/64/212, Harness to Fitzwilliam [16 Feb.]).

In February 1814, Harness felt forced to conclude that the experiment had largely failed. He reported to Earl Fitzwilliam and the Committee that ‘much temporary Relief and mitigation of symptoms’ were observable in the early stages, but that ‘permanent Relief has not been obtained by the Process in these Cases’. In its final published report, the Committee advised that the four ‘Public Patients’ placed with Delahoyde and Lucett had all shown sufficient early signs of benefit from the ‘Process’ as to ‘hold out the most flattering prospects of ultimate Success’, but those hopes were not sustained. However, the Committee acknowledged that the process appeared to have brought ‘complete Success’ with several private patients. They consequently still considered the ‘Discovery’ a ‘most valuable one’, and quite safe if implemented by ‘professional and scientific Men’. Moreover, the ‘Mode of Treatment’ was ‘infinitely less painful to the Feelings of their Friends and relatives’ than the means frequently practised under the ‘old System’ (SCA, 1814: WWM/F/64/191, undated report; WWM/F/64/211, Harness to Fitzwilliam [3 Feb.]; WWM/F/64/212, Harness to Fitzwilliam [16 Feb.]; WWM/F/64/213, Harness to Fitzwilliam [19 Feb.]).

Even before publication of the Committee’s conclusions, the situation was unravelling. Indeed, judging from allegations made by James Lucett in 1815, some dubious transactions had been occurring for a while. Lucett apportioned most of the blame to Charles Delahoyde and Richard Troward. He suggested that, following the receipt of funds from ‘several gentlemen’ in 1813, Delahoyde became ‘elated with the prospect of success which now opened up before him, and dazzled by the splendor [*sic*] of the fortune which seemed to be within his grasp’. He increased his ‘personal establishment’ and his expenditure on ‘carriages and horses, grooms and attendants’. His association with the urbane, calculating Troward, who encouraged the rapid development of the scheme, led to the proliferation of lucrative private patients and relocation from Sion Vale to the larger Ealing House. According to Lucett, Troward and Delahoyde received several thousand pounds in a short space of time, while he was largely excluded from their activities. Nevertheless, the other two men’s financial difficulties became so pressing that they were unable to devote sufficient time and attention to the business. Delahoyde’s ‘extravagance now compelled him to absent himself from home for fear of his creditors’, and he went ‘scampering over the country’. As a consequence, the patients were being neglected, placing the whole venture at risk (Lucett, 1815: 16–27).

In April 1814, a critical piece appeared in the influential *Edinburgh Medical and Surgical Journal*, expressing serious reservations regarding the ‘Process practised by Messrs Delahoyde and Lucett’. The writer could not recommend people travelling down from Scotland, doubting ‘whether any measures would restore them in so short a period as two months’. The Committee was criticised for its limited medical input, and particularly because ‘they keep secret what they know’. The level of Charles Delahoyde’s knowledge was ridiculed, having inferred that ‘the changes of the moon’ had an influence upon ‘mad people’ and also linking ‘the state of the pulse’ to ‘the state of the mind’. The article concluded with a damning indictment: ‘we own that we are apt to consider any opinions void of truth when we find absurdities connected with them’ (*Edinburgh Medical and Surgical Journal*, 1814, 10(April): 250–2). This further added to the damage already inflicted upon the credibility of the ‘process’ and its projectors.

Meanwhile, matters had taken a more serious turn. With creditors closing in, Delahoyde was arrested for debt. However, while on bail, he decamped to Ireland. James Lucett was left to face the consequences, and was shortly afterwards committed to the Fleet Debtors’ Prison. Lucett’s main champion, his loyal wife Olivia, starkly described their plight in a letter to Earl Fitzwilliam in May 1814. She sought his intercession with the Royal Dukes and ‘the other members of the noble Committee in behalf of my self and children left unprotected and near confinement again’. Her husband was in prison ‘for Mr Delahoydes debts he having drawn bills and affixed Mr Lucetts name without his knowledge’, to pay various tradespeople. Their credit had been ‘compleatly ruined’ by Delahoyde’s conduct and ‘the failure of the Concern’. She had ‘been obliged to part with every article in our possession accept [*sic*] household furniture’. Her letter also asserted that underhand arrangements were made by Delahoyde, Troward and another dubious character named Montprivat regarding sale of the secret manuscript copy of the ‘process’ (BPP 1813–14, p. 12; SCA, 1814, WWM/F/64/214, Olivia Lucett to Fitzwilliam [18 May]; Lucett, 1815: 26–31).

The nature of the response by Earl Fitzwilliam, the Royal Dukes and their colleagues to Olivia Lucett’s appeal is not known, but her husband’s release from debtors’ prison followed within a few weeks. By early July James Lucett was back in practice and advertising for custom, citing ‘the continued success attending his process for the restoration of Patients placed under his care’. He remained at Ealing for a few months, but was unable to sustain such a substantial operation. By the end of 1814 he had relocated to smaller premises at Datchet, near Windsor, where he could receive ‘a limited number of patients’ (Lucett, 1815: 29–32; *MP*, 5 July, 21 Dec. 1814). His subsequent career will be considered in a forthcoming second article.

## Postscript

Of all the various actors in the drama, Richard Troward probably came off the worst. He had risked a considerable sum of money in the scheme and, notwithstanding the payments he received for his services, when it collapsed his own fall was particularly heavy. Bankruptcy followed not long afterwards, and he died in October 1815 at the age of 64,^4^ in circumstances that were not recorded. Within a few weeks, his assignees were disposing of the lease of his large, prestigious house in Albemarle Street, along with its lavish contents which included ‘Paintings by esteemed Masters’ (SCA, 1815: WWM/F/64/216, Smith to Dent [10 Mar.]; Dent to Fitzwilliam [27 Apr.]; *MP*, 23 Oct. 1815; *Star*, 18, 27 Nov. 1815; Lucett, 1815: 17–18, 27).

As for Charles Delahoyde, once back in Ireland after having abandoned his debts as well as a wife and two young children, he sought quickly to revive his fortunes. In late 1814 ‘Doctor Delahoyde’ and two questionable associates, including Richard Montprivat who had earlier been named by Mrs Lucett, established Elm Villa, a private lunatic asylum near Dublin. The enterprise collapsed after a few months, but not before Delahoyde had impregnated a vulnerable young female servant named Eliza. With bankruptcy looming, the three men persuaded a gullible local physician, Dr P.P. Middleton, to join them in opening Hanover Park asylum in the town of Carlow. Middleton advanced the money for furniture, equipment and the transfer of patients from Elm Villa (Anon., 1816: 11–13, 17–19, 35–8; Dobbing and Tomkins, 2020).^5^

Delahoyde became resident surgeon at Hanover Park. Its publicity, much of it directly lifted from earlier published material regarding Sion Vale, described the royal patronage and great success achieved with patients using his ‘process’, all mention of James Lucett having been carefully removed (Anon., 1815: i–iv, 1–25). The first patients were admitted to Hanover Park in May 1815. In the summer of 1816, the birth of a baby to a patient named Mrs Hester Hinds precipitated a public scandal. By this time Delahoyde was in the Dublin debtors’ prison, from whence he contrived to convince her outraged husband that the elderly Dr Middleton was the child’s father. At the sometimes raucous trial of Middleton in December 1816, it became clear that ‘Dr Delahoyde’ was at the epicentre of a set of sexual misdemeanours involving himself as well as various staff members and patients, including Mrs Hinds and Eliza from Elm Park. He had also evidently been blackmailing the hapless Dr Middleton (*Bell’s Weekly Messenger*, 22 Dec. 1816; *Dublin Evening Post*, 16 Nov., 17 Dec. 1816; Anon., 1816: 4–6, 12–18, 20–26, 35–68; Dobbing and Tomkins, 2020: 4–6). By that time Delahoyde had emerged from jail, left Hanover Park and attempted to establish himself in private practice in Dublin, once again puffing his therapeutic triumphs in London (*Dublin Evening Post*, 28 Sep.–2 Nov. 1816). However, the disgrace arising from the scandal destroyed what was left of his professional credibility. At some point he returned to London and subsequently entered into cohabitation with Mrs Letitia Shadwell, a vulnerable wealthy widow who had a history of serious mental illness. Little else was heard of Delahoyde before his untimely death in 1823 (*The Times*, 9 Feb. 1830).

[Part 2 will be published in the next issue of *History of Psychiatry*.]

## Notes

1. The order used in citations follows the order of the reference lists at the end: archival sources (using the abbreviations given in the entries listed); newspapers; other publications.

2. Dr Robert Darling Willis was a son of Dr Francis Willis.

3. I am grateful to Dr Catherine Beck for providing the naval references.

4. Find A Grave Memorials; accessed (18 Nov. 2021) at: www.findagrave.com/memorial/180548305/richard-ironnmonger-troward

5. I am grateful to Dr Alice Mauger for locating items published in Ireland.
